# The *Non-Coding RNA* Journal Club: Highlights on Recent Papers—10

**DOI:** 10.3390/ncrna8010003

**Published:** 2022-01-10

**Authors:** Jairo A. Pinzon Cortes, Assam El-Osta, Giulia Fontemaggi, Nicholas Delihas, Katsuki Miyazaki, Ajay Goel, Mira Brazane, Clément Carré, Paola Dama, Salih Bayraktar, Leandro Castellano, Francisco J. Enguita, Tijana Mitic, Andrea Caporali, André P. Gerber, Nicola Amodio

**Affiliations:** 1 Epigenetics in Human Health and Disease, Department of Diabetes, Central Clinical School, Monash University, Melbourne, VIC 3004, Australia; jairo.pinzoncortes@monash.edu; 2 Oncogenomic and Epigenetic Unit, IRCCS Regina Elena National Cancer Institute, Via Elio Chianesi 53, 00144 Rome, Italy; 3 Department of Microbiology and Immunology, Renaissance School of Medicine, Stony Brook University, Stony Brook, New York, NY 11794, USA; 4 Department of Molecular Diagnostics and Experimental Therapeutics, Beckman Research Institute of City of Hope, Biomedical Research Center, Monrovia, CA 91016, USA; kmiyazaki@coh.org; 5 Department of Surgery, Tokushima University, Tokushima 7708503, Japan; 6 City of Hope Comprehensive Cancer Center, Duarte, CA 91010, USA; 7 Transgenerational Epigenetics & Small RNA Biology, Sorbonne Université, CNRS, Laboratoire Biologie du Développement, Institut de Biologie Paris-Seine, UMR7622, 75005 Paris, France; 8 School of Life Sciences, University of Sussex, Falmer, Brighton BN1 9QG, UK; 9 Department of Surgery and Cancer, Imperial Centre for Translational and Experimental Medicine (ICTEM), Imperial College London, London W12 0NN, UK; 10 Instituto de Medicina Molecular João Lobo Antunes, Faculdade de Medicina, Universidade de Lisboa, Av. Prof. Egas Moniz, 1649-028 Lisbon, Portugal; 11 University/British Heart Foundation Centre for Cardiovascular Science, The Queen’s Medical Research Institute, University of Edinburgh, Edinburgh EH16 4TJ, UK; acaporal@exseed.ed.ac.uk; 12 Department of Microbial Sciences, School of Biosciences and Medicine, Faculty of Health and Medical Sciences, University of Surrey, Guildford GU2 7XH, UK; 13 Department of Experimental and Clinical Medicine, Magna Graecia University of Catanzaro, 88100 Catanzaro, Italy

## 1. Introduction

We are delighted to share with you our seventh Journal Club and highlight some of the most interesting papers published recently. We hope to keep you up-to-date with non-coding RNA research works that are outside your study area. The *Non-Coding RNA* Scientific Board wishes you an exciting and fruitful read.

## 2. Identifying Novel miRNAs as Predictive Biomarkers of End Stage Kidney Disease (ESKD) in Diabetes

### Highlight by Jairo A. Pinzon Cortes and Assam El-Osta

In a finding that has elated and perplexed researchers, a recent study has shown circulating determinants as predictive biomarkers for end stage kidney disease (ESKD). A key objective—because not all diabetic patients progress identically—is the early prediction of diabetic kidney disease (DKD). To address this unmet need, the study has assessed the expression of circulating miRNAs and proteins in peripheral blood from individuals with DKD (type 1 and type 2 diabetes) from the Joslin Kidney Study (88% of European–American ancestry) and the Pima Indian Kidney Study (100% of Indigenous–American ancestry) [1]. Statistically, 17 miRNAs were found to be significant and differentially expressed, pointing towards networks that influence the Axonal Guidance Pathway (AGP) and Ras signaling. Furthermore, proteomic analyses using SOMAscan and Olink identified 6 proteins that converge on the AGP pathway that were associated with the onset of ESKD. The study findings suggest these proteins could represent biomarkers for the early detection of DKD. The predictive performance of the AGP proteins were calculated as hazard ratios for the association with ESKD at 7, 10 and 15 years of follow-up that ranged between 1.42 and 2.15. Surprisingly, close scrutiny of kidney tissue showed no correlation with mRNA expression or lesion severity, suggesting the biomarkers are likely to originate from outside the kidney. Following the identification of specific miRNAs and peptides, the challenge now is to understand their function in the progression of DKD. Facing the complex problem of predicting diabetic complications, the novel biomarker assay using blood is considered safe, cost-effective and quick. This may represent a major advance that could serve as a clinical aid in the prediction of ESKD. While there is a strong correspondence for circulating determinants in the Joslin and Pima cohorts, further studies are required to validate these findings in different DKD populations. Granted the study has a modest group size, future studies considering statistical design and power estimates will be a clinical requirement.

## 3. The Structural Characteristics of lncRNA MALAT1 Determine Its Ability to Interact with the RNA-Binding Protein NONO

### Highlight by Giulia Fontemaggi

MALAT1 is one of the most abundant and highly conserved lncRNAs and contributes to various features of malignancy, as proliferation and migration. Subnuclear localization and activity of MALAT1 are controlled by various protein factors. The N6-methyladenosine (m6A) writer METTL3 for example methylates MALAT1 lncRNA leading to its stabilization, while the m6A reader YTHDC1 binds to m6A-modified MALAT1 and regulates the composition/function of nuclear speckles, strongly contributing to the metastatic ability of cancer cells. Mou and colleagues have now reported that MALAT1 activity is not only controlled by epitranscriptomic modifications but also by complex RNA structures termed RNA G-quadruplexes (rG4) [2]. rG4 take part in cancer development through numerous biological functions, such as the control of RNA localization and translation. Thus far, rG4 have been mainly characterized in protein-coding mRNAs, while information about its presence and function in lncRNAs is very limited. This study reveals that rG4s in MALAT1 can interact with the RNA binding protein (RBP) NONO with high specificity and affinity. RBP NONO has been reported to control proliferation and resistance to chemotherapy in cancer cells. Although this study does not present specific functional implications for cancer, the identification of this structural feature controlling MALAT1-NONO interaction opens to the interesting possibility that rG4 represents a main layer of control of MALAT1 activity in cancer cells and merits further cancer-focused investigation.

## 4. The Human Long Intergenic Non-Coding RNA Gene *linc-UR-UB*, Simple Origins but Complex Functions

### Highlight by Nicholas Delihas

How new genes are formed is currently a major topic of interest, for example, see a special issue of *Genes* (ISSN 2073-4425), “How Do New Genes Originate and Evolve?” (https://www.mdpi.com/journal/genes/special_issues/genes_originate_evolve (accessed on 5 January 2022)). This special issue belongs to the section “Population and Evolutionary Genetics and Genomics”. The interesting and multifaceted paper by Rubino et al [3] shows the birth of the human long intergenic non-coding RNA gene, *linc-UR-UB.* There is beauty in the simplicity of formation of this gene; it involves a transcriptional read through from an existing gene to a small functional unit present in the downstream non-coding DNA region of the gene, thus creating a new gene and new function [3,4]. Transcriptional read throughs are not uncommon, so we may expect to see more genes that are formed in this manner. The proposed *linc-UR-UB* transcript functions are fascinating: a lincRNA that may regulate the immune system by serving as a “sponge” for miRNAs, where the miRNAs themselves regulate expression of the human ubiquitin-specific peptidase 18 and thus regulate interferon [3]. The indirect evidence for the “sponge” function is strong and is consistent with criteria presented in the *non-coding RNA* Journal Club—9, abstract, “5. No Co-expression, No Sponging”, highlighted by Laura Poliseno [5].

## 5. Hypoxia-Inducible Exosomes from Colorectal Cancer (CRC) Cells Contribute towards Premetastatic Niche Formation in Liver

### Highlight by Katsuki Miyazaki and Ajay Goel

Liver is one of the most common distant metastasis sites and liver metastasis is one of the major causes of death among patients with colorectal cancer (CRC). Not surprisingly, the treatment and prevention of liver metastasis remains an urgent need that must be addressed in clinical practice of CRC. There are two major factors responsible for metastasis formation in CRC—the premetastatic niche and the tumor microenvironment. In a recent study, Sun et al. reported that hypoxic tumor microenvironment boosted the release of exosomes from cancer cells, comprising of miR-135a-5p and, more specifically, exosomal miR-135a-5p contributed to liver-specific premetastatic niche formation [6].

In the study, the authors noted that the expression of both tumoral and serum miR-135b-5p was enriched in patients with CRC liver metastasis. Furthermore, their invitro and in vivo experiments revealed that CRC cells released miR-135a-5p enriched exosomes in hypoxic environment. Circulating miR-135a-5p enriched exosome could be phagocytosed by Kupffer cells, and exosomal miR-135a-5p initiated premetastatic niche formation in liver by promoting cell adhesion through LATS2-YAP-MMP7 axis. Exosomal miR-135a-5p also has immunosuppressive effect through CD30–TRAF2–NF-κB signal.

These findings are truly promising for clinical practice of CRC as these suggest that inhibition of exosomal miR-135a-5p might serve as a potential target for treatment and prevention of CRC liver metastasis.

## 6. To Be or Not to Be Coding, That Is the Readthrough Question

### Highlight by Mira Brazane and Clément Carré

Translational readthrough (TR) generates proteins with extended C-termini that can modify protein function. TR recoding occurs when the ribosome decodes a stop codon as a sense codon, resulting in two protein isoforms synthesized from the same mRNA. TR is utilized by viruses, yeast and higher eukaryotes such as *Drosophila*, mosquitos and mammals. TR has been identified in several eukaryotic organisms’ tissues with a predominance in neuronal cells. However, the precise molecular actors that control those TR events in a tissue specific manner remains mysterious. Karki et al. recently quantified TR of candidate genes in *Drosophila melanogaster* and characterized in detail the regulation of TR of the transcription factor Traffic jam (Tj) [7]. They showed that the TR-generated Tj protein isoform is expressed in some neural cells of the central nervous system and is excluded from the somatic cells of gonads in contrast to non-TR Tj isoform. The authors identify a tissue-specific distribution of a release factor (RF) splice variant, eRF1H, that plays a critical role in increasing TR, providing an attractive explanation behind elevated incidence of TR of leaky stop codon in brain tissue. Finally, the authors show that near cognate tRNAs to UGA stop codons known to favor TR are also involved, increasing the TR capacity in brain tissue where they are more abundant. The authors conclude that selective TR may serve to enrich the diversity of the neuronal proteome.

## 7. Phase Separation Can Mediate Interactions between lncRNAs and RNA Binding Proteins

### Highlight by Paola Dama, Salih Bayraktar and Leandro Castellano

The article by M.E. Elguindy and J.T. Mendell on *Nature* [8], entitled: “NORAD-induced Pumilio phase separation is necessary for genome instability,” describes how NORAD lncRNA exploits phase separation to amplify its ability to interact and regulate their RNA-binding protein (RBP) targets; PUM1 and PUM2 (PUMs). NORAD is a highly conserved cytoplasmatic lncRNA induced by DNA damage. Mechanistically, it binds to PUMs to negatively regulate their activities for genome stability modulation in mammals. PUMs are post-transcriptional repressors that bind to consensus sequences known as Pumilio response elements (PREs) in mRNA targets to stimulate their degradation. In the absence of NORAD, hyperactive PUMs repress key mitotic mRNAs inducing mitotic errors. Previously, it was unclear how NORAD could sequester a significant fraction of PUMs to outcompete all their targets during the regulation of genome maintenance. However, they reveal that NORAD and PUMs colocalize in liquid-like condensates called NORAD-Pumilio (NP) bodies, and phase separation is critical for concentrating enough PUMs in these structures. Interactions via intrinsically disordered regions (IDRs) between PUMs also play a central role in droplet formation and regulation of genome maintenance. This study proposes that RNA-driven phase separation is a new mechanism of RBP regulation and indicates that phase separation may be a broad mechanism used by lncRNA to regulate cellular processes.

## 8. RNA Structural Landscape of Dicer Substrates

### Highlight by Francisco J. Enguita

Dicer is a RNA endonuclease involved in the biogenesis of miRNAs and other regulatory non-coding RNAs. The characteristic structural shape of Dicer confers the property of acting as a “molecular ruler” that measures and cleave double-stranded RNAs (dsRNA) generating products with specific sizes. The manuscript by Luo and co-workers, published in *Nature Communications*, describes a very interesting approach to study the substrate specificity of Dicer by combining a new RNA structure-probing method (icSHAPE-MaP, an approach combining in vivo selective 2′-hydroxyl acylation with 2-methylnicotinic acid imidazolide-azide, NAI-N3, and mutational profiling to probe intact RNA structures), together with an antibody pull-down of the enzyme [9]. The authors proposed a structural landscape for Dicer targets that depends on the size of the terminal loop of the dsRNA substrate. The results allowed them to stratify the analysed substrates into three categories that are dominated by different species of ncRNAs. The miRNAs typically harbor bigger terminal loops (9 nt), whereas other ncRNA substrates such as tRNAs or snoRNAs have smaller terminal loops (around 7 nt) surrounded by a loose stem. The paper is completed by a very detailed experimental validation of different Dicer substrates, concluding that the enzyme can process a great variety of substrates with specific affinities. Overall, the published data constitutes an excellent approach for the characterization of Dicer specificities and also for the dissection of the molecular mechanisms involved in the generation of trans-acting RNAs from substrates different to the precursor miRNAs.

## 9. RNA-RNA Interactome Helps Understand the Recruitment and Activation of PRC2 by lncRNAs

### Highlight by Tijana Mitic and Andrea Caporali

The transcriptional repressor Polycomb Repressive Complex 2 (PRC2) is a critical epigenetic regulator in various biological contexts. Beyond its chromatin binding capacity, PRC2 has attracted enormous interest for its interactions with RNA and many long non-coding RNAs (lncRNAs). Conversely, RNA can act as a catalytic inhibitor of PRC2 activity, although it has remained unclear how this inhibition is relieved in the contexts when lnRNA is present near chromatin region, without prior H3K27me3 being deposited.

The study by Balas et al. describes a model for HOTAIR lncRNA and the RNA binding protein hnRNP B1 engaging in multivalent protein–RNA interactions. The inhibitory effects of HOTAIR on the PRC2 catalytic activity can be overcome by specific intermolecular RNA–RNA interactions of HOTAIR with its targets [10]. There are multiple domains that hnRNP B1 uses to engage with HOTAIR regions in a way that can bridge this lncRNA to the RNA of a target gene. Specifically, the secondary structure of HOTAIR is remodelled by hnRNP B1, making a more favourable interaction with its target, JAM2. In turn, the formation of duplex between HOTAIR and JAM2 reduces the HOTAIR ability to inhibit PRC2. In this proposed model, hnRNP B1 must dissociate from the complex before HOTAIR inhibitory effects on PRC2 are relieved, and PRC2 activity is promoted. This intrinsic switch could be a critical mechanism in understanding how lncRNAs drive PRC2 activity also in a disease setting. In such contexts, transcriptome-wide immunoprecipitation analyses with RNA–RNA interactome, such as formaldehyde cross-linking, ligation and sequencing of RNA hybrid (FLASH) approach, could generate nucleotide specific sites of PRC2-lncRNA interaction. The secondary structure obtained through RNA–RNA interactome could give insight into the nature of PRC2 engagement with RNA; simultaneous PRC2 activity at chromatin loci in the vicinity could be assessed.

## 10. SARS-CoV-2 Expresses a MicroRNA That Could Repress Interferon-Related Host Genes

### Highlight by André P. Gerber

Severe acute respiratory syndrome coronavirus 2 (SARS-CoV-2) is the causative agent of coronavirus disease (COVID-19), which is of great health concern worldwide.

In a recent paper published in *Proceedings of the National Academia of Sciences*, Joan Steitz’s laboratory investigated the impact of SARS-CoV-2 infection on host microRNA populations in human lung-derived cell lines and in nasopharyngeal swabs from SARS-CoV-2 infected individuals [11]. While no significant impact of SARS-CoV-2 infection on host-cell miRNA populations was observed, a miRNA-like small RNA, called CoV2-miR-O7a, expressed from an evolutionarily conserved hairpin within the ORF7a sequence of SARS-CoV-2 was identified. CoV2-miR-O7a is associated with Argonaute proteins acting in the RNA interference pathway; and a computational approach identified putative mRNA targets for CoV2-miR-O7a, including Basic Leucine Zipper ATF-Like Transcription Factor 2 (BATF2) which participates in interferon signalling. BATF2 mRNA levels were significantly downregulated in CoV2-miR-O7a transfected cells, and production of CoV2-miR-O7a depends on the cellular machinery, albeit independent of DROSHA involved in early precursor-miRNA processing.

The study indicates that CoV2-miR-O7a could contribute to SARS-CoV-2 pathogenesis. It aligns with a related study from the Cecere lab that confirms the existence of this viral miRNA-like molecules, possibly regulating host genes to evade interferon-mediated immune response [12].

## 11. Long Non-Coding RNAs May Hide Oncogenic Protein-Coding RNAs!

### Highlight by Nicola Amodio

Long non-coding RNAs (lncRNAs) represent the greatest part of the human transcriptome, mainly acting as microRNA sponges, transcriptional scaffolds or epigenetic modulators; only recently, it has become apparent that open reading frames (ORFs) can be found even within lncRNAs transcripts. In a recent paper published on *The Journal of Clinical Investigation*, the authors characterize an ORF within the LINC00467 sequence, encoding for a micropeptide named ASAP (ATP synthase-associated peptide) for its ability to interact with ATP synthase subunits α and γ (ATP5A and ATP5C) [13]. By elegant mechanistic studies, the authors show that LINC00467 oncogenic activity in colorectal cancer (CRC) is strictly dependent on the production of ASAP micropeptide, that promotes ATP synthase assembly and, in turn, enhances mitochondrial ATP production. By using CRC preclinical models, the authors show that ASAP promotes in vitro and in vivo tumor growth, and its tumorigenic potential is dependent on OXPHOS triggering and ATP release. Notably, ASAP expression is upregulated in CRC biopsies with respect to normal colonic cells, and portends poor prognosis of patients. This work reminds us the plethora of undisclosed functions of lncRNAs, shifting from the old “transcriptional noise” theory of the lncRNome to a novel “translational noise” perspective; moreover, it underscores the relevance of OXPHOS-based metabolic alterations behind CRC pathogenesis. Although the therapeutic potential of ASAP targeting by CRISPR-Cas9 is noteworthy, detailed analysis of ASAP interaction with ATP synthase through biophysical approaches must be carried out to develop ASAP inhibitory compounds, thus making it a real druggable target in human cancer.
